# Application of Amino-Functionalized Metal–Organic Framework UiO-66-NH_2_ in the Remediation of Multi-Metal-Contaminated Soil in Mining Areas

**DOI:** 10.3390/toxics14060462

**Published:** 2026-05-25

**Authors:** Jie Yang, Yulong Yan, Hang Chen, Wanzi Li, Rui Zuo

**Affiliations:** 1 College of Water Sciences, Beijing Normal University, Beijing 100875, China; yangjie@bnu.edu.cn (J.Y.); zr@bnu.edu.cn (R.Z.); 2 Engineering Research Center of Groundwater Pollution Control and Remediation, Ministry of Education, Beijing 100875, China

**Keywords:** metal–organic frameworks, UiO-66-NH_2_, heavy metal contamination, soil amendments, microbial community

## Abstract

Traditional adsorbents, such as clay minerals, mainly target metal cations, which limits their effectiveness in mining soils where multiple metals and oxyanionic species coexist. To address this limitation, we evaluated amino-functionalized UiO-66-NH_2_ as a stabilizing amendment capable of immobilizing both anionic and cationic metal species. This study evaluated its stabilization performance for vanadium (V), chromium (Cr), nickel (Ni), and zinc (Zn) in typical mining soils. Soil samples were amended with 1%, 2%, and 3% dosages and incubated for 50 days to systematically analyze leaching behavior, speciation transformation, and microbial community responses. At 50 days, the 1% UiO-66-NH_2_ treatment reduced the leaching concentrations of V, Cr, Ni, and Zn by 90.42%, 59.72%, 90.12%, and 90.71%, respectively. Although Cr showed its highest reduction efficiency under the 3% treatment, the 1% dosage provided a practical compromise for multi-metal stabilization rather than a universal optimum. The stabilization process was primarily driven by surface complexation and ion exchange, which facilitated the transformation of metals into stable oxidizable forms. The amendment increased soil organic matter (SOM) and cation exchange capacity (CEC), triggering competitive adsorption and increasing the mobility of Zn and Ni. Microbial profiling revealed a successional shift toward resilient taxa, specifically the proliferation of Actinomycetota and *Arthrobacter*. These findings established that a 1% UiO-66-NH_2_ amendment provides a robust and ecologically compatible strategy for the sustainable remediation of complex mining-impacted environments.

## 1. Introduction

With rapid industrialization and mining activities, soil contamination by heavy metals has become a serious environmental concern. In mining-impacted areas, soils are often contaminated by multiple metals simultaneously rather than by a single element [1,2,3]. Such multi-metal contamination is more difficult to remediate because coexisting metals may differ in ionic form, mobility, and chemical reactivity. Interactions among coexisting metals can also lead to inconsistent stabilization efficiencies or even promote the mobility of certain metals [4,5].

Stabilization is widely used for the remediation of heavy-metal-contaminated soils because it can reduce metal mobility and bioavailability through adsorption, ion exchange, complexation, precipitation, and redox-related processes [6,7,8,9,10]. However, conventional stabilizers, such as clay minerals, are generally more effective for metal cations and may show limited performance in complex systems where cationic metals and oxyanionic species coexist [11]. Therefore, amendments capable of simultaneously immobilizing different metal species are needed for the remediation of multi-metal-contaminated soils.

Metal–organic frameworks (MOFs) have attracted increasing attention as adsorbents because of their high surface area, tunable pore structures, and abundant functional sites [12,13,14,15,16]. Among them, zirconium-based UiO materials exhibit relatively high chemical and thermal stability, making them promising candidates for environmental applications [17,18]. Functionalized UiO-66 materials have been reported to enhance heavy-metal adsorption through surface complexation, electrostatic interaction, and ion exchange [19,20,21,22,23]. In particular, amino-functionalized UiO-66-NH_2_ contains surface –NH_2_ groups that may contribute to the immobilization of both anionic and cationic metal species, suggesting its potential for treating multi-metal-contaminated soils.

In addition to metal immobilization, the ecological response of soil after amendment application should be considered. Soil microorganisms are sensitive indicators of soil environmental quality and play important roles in nutrient cycling and ecosystem recovery [24]. Stabilization amendments may alter soil physicochemical properties, such as soil organic matter and cation exchange capacity, thereby affecting microbial community structure. However, most studies on UiO-66 and its derivatives have focused on aqueous heavy-metal removal, while their effects on metal speciation and microbial communities in complex soil matrices remain insufficiently understood [22,23,24].

In this study, UiO-66-NH_2_ was applied as a stabilizing amendment to mining-area soil contaminated with V, Cr, Ni, and Zn. The objectives were to: (i) evaluate the effects of different UiO-66-NH_2_ dosages on metal leaching behavior; (ii) clarify the transformation of metal chemical fractions during stabilization; (iii) assess changes in soil physicochemical properties, including SOM and CEC; and (iv) investigate microbial community responses after amendment application. This study provides a laboratory-based evaluation of UiO-66-NH_2_ for the stabilization of multi-metal-contaminated soil and supports the development of functional amendments for complex mining-impacted environments.

## 2. Experimental Section

### 2.1. Sample Collection and Analysis

Heavy-metal-contaminated soil was collected from five sites at a depth of 0–20 cm in the Zhujiabaobao mining field, Pangang Group concentrator (Panzhihua, China), vanadium and titanium high-tech industrial development zone, Xiaobaoding (Panzhihua, China), and Huashan mine in Panzhihua city, Sichuan Province. After collection, the soil samples were naturally air-dried at room temperature in a ventilated laboratory, manually cleared of stones, plant roots, leaves, and other visible impurities, homogenized thoroughly, passed through 20–100 mesh sieves, and stored in sealed polyethylene bags before further analysis.

ICP-AES results showed that the average concentrations of V, Cr, Ni, and Zn in the study-area soil exceeded the background values for soils in Sichuan Province, indicating multi-metal contamination dominated by V and Cr (Table S1).

Soil pH was measured at each incubation time point using a pH meter in a soil–water suspension at a soil-to-water ratio of 1:2.5 (*w*/*v*). Soil organic matter (SOM) was determined using the potassium dichromate external heating method. Cation exchange capacity (CEC) was measured by the cobalt (III) chloride extraction–spectrophotometric method. Leaching toxicity was determined according to the “Toxicity Leaching Method for Solid Waste—Horizontal Oscillation Method” [25]. The speciation of V and its associated heavy metals in the soil (weak acid extractable, reducible, oxidizable, and residual fractions) was analyzed using the BCR sequential extraction method.

The synthesized UiO-66-NH_2_ was characterized using X-ray diffraction (XRD; Rigaku D/MAX-2500, Rigaku, Tokyo, Japan) to determine the crystal structure, Fourier transform infrared spectroscopy (FTIR; Nicolet iS5, Thermo Fisher Scientific, Waltham, MA, USA) to identify surface functional groups, scanning electron microscopy coupled with energy-dispersive X-ray spectroscopy (SEM-EDS; Merlin Compact, Carl Zeiss, Oberkochen, Germany, and X-Max, Oxford Instruments, Abingdon, UK) to observe morphology and elemental composition, and nitrogen physisorption analysis (ASAP 2460, Micromeritics, Norcross, GA, USA) to characterize the specific surface area and pore structure.

### 2.2. Synthesis of UiO-66-NH_2_ and Soil Stabilization Design

UiO-66-NH_2_ was synthesized using an ambient-pressure reflux system. Zirconium chloride (756 mg) and 2-aminoterephthalic acid (584 mg) were dissolved in a mixed solvent of acetic acid (57.74 g) and N, N-dimethylformamide (340 mL) [26]. After ultrasonic homogenization to ensure complete dispersion of the precursors, the solution was transferred into a flask equipped with a reflux condenser. The reaction was conducted at 120 °C for 6 h under magnetic stirring at 400 rpm. Upon completion, the mixture was centrifuged and washed to remove residual solvent and unreacted precursors. The obtained solid was then dried under vacuum at 60 °C for 12 h, yielding the UiO-66-NH_2_ series of materials.

For the soil stabilization experiment, 20 g of dried soil was placed into 200 mL beakers and amended with UiO-66-NH_2_ at 1%, 2%, and 3% based on dry soil weight. The untreated contaminated soil collected before UiO-66-NH_2_ addition was defined as the initial untreated baseline sample. This time-zero sample was used to characterize the initial leaching concentrations and chemical fractions of V, Cr, Ni, and Zn before stabilization, rather than as an independently incubated time-matched control treatment. The soil and amendment were thoroughly mixed, and deionized water was added to maintain relatively stable soil moisture during incubation. Every 10 days, leaching toxicity tests and BCR sequential extraction experiments were conducted to evaluate changes in V, Cr, Ni, and Zn after UiO-66-NH_2_ treatment. Soil physicochemical properties were also measured to evaluate the effects of UiO-66-NH_2_ amendment on the soil environment. The stabilization efficiency was used to evaluate the stabilization performance of heavy metals such as V and Cr, and was calculated using the following equation:

(1) $$R_{st}=\frac{C_{1}-C_{2}}{C_{1}}\times 100\%$$

where $R_{st}$ represents the stabilization efficiency (%); and $C_{1}$ and $C_{2}$ are the leaching concentrations of the metals before and after stabilization (mg/L), respectively.

To ensure the reliability and accuracy of the experimental results, all experiments were performed in triplicate, and the data were analyzed and plotted as mean ± standard error (SE). In the soil stabilization experiments, the recovery rates of the different heavy metal fractions were maintained within the range of 95% to 105%.

### 2.3. Soil DNA Extraction and Data Analysis

16S rRNA gene amplicon sequencing was performed to investigate microbial community structure, co-occurrence patterns, and community assembly [27]. For microbial community analysis, each treatment group was represented by three independent soil samples. Total genomic DNA was isolated from soil samples using the E.Z.N.A.^®^ Soil DNA Kit (Omega Bio-tek, Norcross, GA, USA) following the manufacturer’s protocol. The V3-V4 hypervariable regions were targeted for amplification using the primer set 338F (5′-ACTCCTACGGGAGGCAGCAG-3′) and 806R (5′-GGACTACHVGGGTWTCTAAT-3′).

The PCR thermal profile consisted of an initial denaturation at 95 °C for 3 min, followed by 30 cycles of 95 °C (30 s), 50 °C (30 s), and 72 °C (60 s), with a final 5 min extension at 72 °C. The resulting amplicons were verified via 2% agarose gel electrophoresis, purified using the AxyPrep DNA Gel Extraction Kit (AXYGEN, New York, NY, USA), and standardized with QuantiFluor™-ST (Promega, Madison, WI, USA). Sequencing libraries, constructed using the NEBNext^®^ Ultra™ DNA Library Prep Kit (NEB, Ipswich, MA, USA), were quality-validated on a Qubit^®^ 2.0 Fluorometer and Agilent 2100 Bioanalyzer before being sequenced on the Illumina MiSeq platform (250 bp/300 bp paired-end). Raw sequences were clustered into operational taxonomic units (OTUs) at a 97% similarity threshold via QIIME2 [28]. Bioinformatics analysis was primarily performed on the Majorbio Cloud Platform (Shanghai Majorbio Bio-pharm Technology Co., Ltd., Shanghai, China, 2020), with subsequent statistical evaluations and data visualization implemented using R (v4.1.3) within the RStudio environment. The microbial α-diversity indices, including Richness, Shannon, and Chao1, were used to describe the observed variation in microbial community diversity among treatments. The microbial community results were interpreted as descriptive trends unless otherwise stated.

## 3. Results and Discussion

### 3.1. Characterization Results of the UiO-66-NH_2_ Material

**Figure 1:**
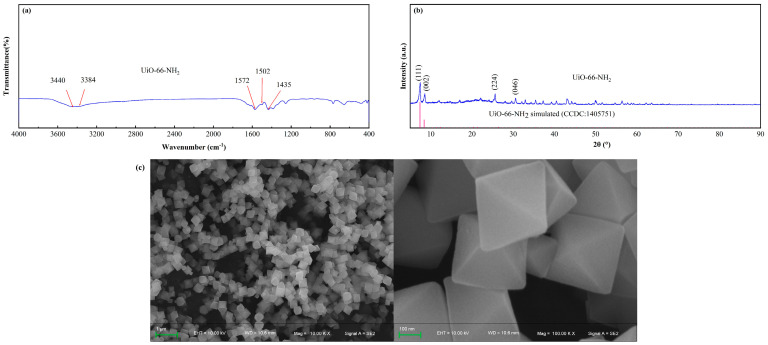
(**a**) FTIR spectra scanning, (**b**) XRD and (**c**) Scanning electron microscopy.

The morphology and structure of the metal–organic framework material UiO-66-NH_2_ observed by scanning electron microscopy are shown in Figure 1c. Overall, UiO-66-NH_2_ particles were relatively uniform in size and exhibited a well-defined octahedral morphology, indicating the typical morphological features of the UiO-66 series [29]. The surface of UiO-66-NH_2_ is smooth and compact, with particle sizes of approximately 300 nm. The particles showed good dispersion and abundant porous structures. The excellent dispersion and abundant pore structure provide ample sites and space for the adsorption of heavy metals in the soil.

As shown in the infrared spectrum in Figure 1a, two absorption peaks in the high-frequency region correspond to the N–H stretching vibration peaks in the NH_2_ group of the organic ligand. Analysis revealed characteristic bands related to framework vibrations in the low-frequency region, including N–H bending vibrations and the characteristic C–N stretching absorption peak. Comparison with the characteristic spectrum of the amino group confirms the presence of amino functional groups. Additionally, characteristic carboxyl group peaks and benzene ring stretching vibration peaks were identified in the spectrum, further confirming the presence of amino, carboxyl, and benzene ring functional groups in the material. The amino groups can be protonated to form –NH_3_^+^ sites that electrostatically attract oxyanionic metal species, such as Cr(VI), and can also coordinate with metal cations through the lone-pair electrons of nitrogen [30]. The benzene ring improves the adsorption of metal cations via cation–π interactions, and the high surface area provides abundant active sites, contributing to the excellent adsorption performance of UiO-66-NH_2_ [17].

The XRD characterization results in Figure 1b confirm that the characteristic diffraction peaks of the synthesized UiO-66-NH_2_ closely match the simulated diffraction pattern of its single-crystal structure (CCDC 1405751), and the peak positions align with the crystallographic features of UiO-66, providing strong evidence for the material’s expected crystal structure [31]. The diffraction peaks are well defined, indicating that the synthesized UiO-66-NH_2_ has a high degree of crystallinity, which suggests that the material possesses a highly ordered lattice structure. This structure ensures long-term stability in complex soil environments. Additionally, the integrity of the crystallization maintains the uniformity of the micropore channels, preventing pore collapse, preserving high surface area, and providing ample adsorption sites [29].

### 3.2. Effects of Stabilization on the Leaching Concentrations and Chemical Speciation of Heavy Metals

#### 3.2.1. Leaching Concentration

**Figure 2:**
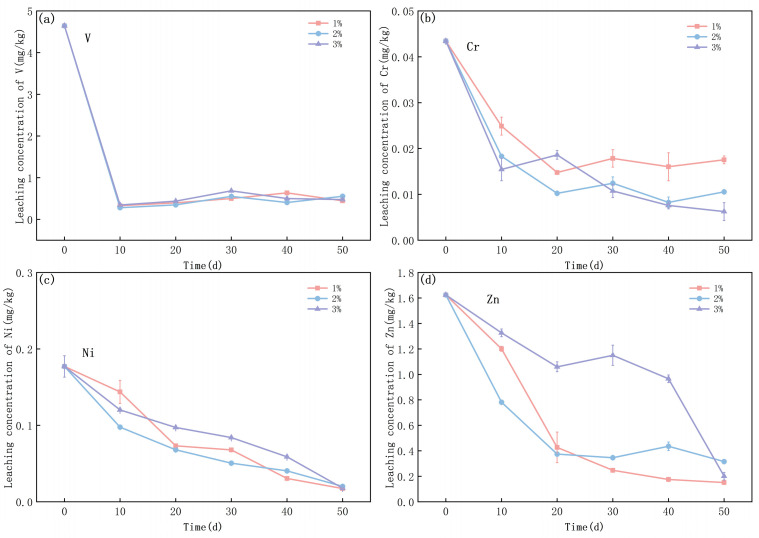
Changes in the leaching concentrations of V (**a**), Cr (**b**), Ni (**c**), and Zn (**d**) over time after UiO-66-NH_2_ amendment. The values at day 0 represent the initial untreated baseline before amendment addition.

**Figure 3:**
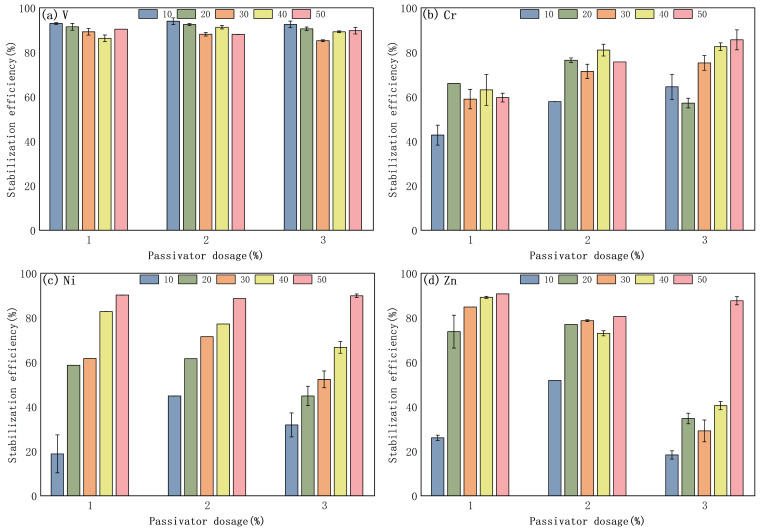
Stabilization efficiencies of V (**a**), Cr (**b**), Ni (**c**), and Zn (**d**) over time under different UiO-66-NH_2_ dosages. Stabilization efficiencies were calculated relative to the initial untreated baseline at day 0. Error bars represent the standard error of the mean (SE, *n* = 3).

The effects of adding different doses of the UiO-66-NH_2_ amendment on the leaching concentration and stabilization efficiency of V and its associated heavy metals in the soil after stabilization with UiO-66-NH_2_ are shown in Figure 2 and Figure 3. The initial leaching concentrations of V, Cr, Ni, and Zn in the untreated contaminated soil were 4.645, 0.043, 0.177, and 1.624 mg/kg, respectively (Tables S3–S6). These values represent the time-zero baseline before UiO-66-NH_2_ amendment and were used to calculate the percentage reduction in metal leaching after stabilization.

The leaching behavior of V depended on both incubation time and UiO-66-NH_2_ dosage. At addition rates of 1–3%, the leaching concentration decreases sharply from 4.645 mg/kg to 0.328–0.345 mg/kg within 10 days, with the passivation efficiency exceeding 92%. However, as the stabilization time increased to 50 days, the leaching concentration rebounded systematically (0.445–0.554 mg/kg), and the efficiency decreased to 88–90% (Tables S7–S10). Notably, the stabilization effect for the three addition rates is very similar during the early stage (10 days), with the 1% addition yielding slightly better long-term stability.

On the other hand, the leaching response of Cr is markedly different (Figure 2b). Its leaching concentration decreased as the addition amount increased. At 1% addition, the concentration decreased from 0.0435 mg/kg to 0.0175 mg/kg within 30 days, achieving an efficiency of 59.12%, but by 50 days, the efficiency increased only slightly to 59.72%. At 3% addition, the concentration sharply decreased to 0.0062 mg/kg, with an efficiency of 75.31%, and the efficiency increased to 85.71% at 50 days. These results indicate that Cr stabilization was more dose-dependent than that of V, Ni, and Zn, and that the highest Cr reduction efficiency was obtained under the 3% treatment rather than the 1% treatment.

Both Ni and Zn exhibit similar dose-threshold effects. Both metals achieve efficient stabilization at 1–2% addition. The Ni leaching concentration decreased from 0.1771 mg/kg to 0.0174–0.0202 mg/kg within 40 days, with an efficiency of 88–90% at 50 days, whereas the efficiency for the 3% addition group declines to 89.89% (Figure 2c and Figure 3c). For Zn, at the 1% dosage, the leaching concentration decreased from 1.201 mg/kg to 0.151 mg/kg, achieving an efficiency of 90.71% (Figure 2d and Figure 3d). At 2–3% addition, the efficiency decreased to 80–87%, with the 2% treatment showed higher leaching concentrations than the 1% treatment.

#### 3.2.2. Speciation

**Figure 4:**
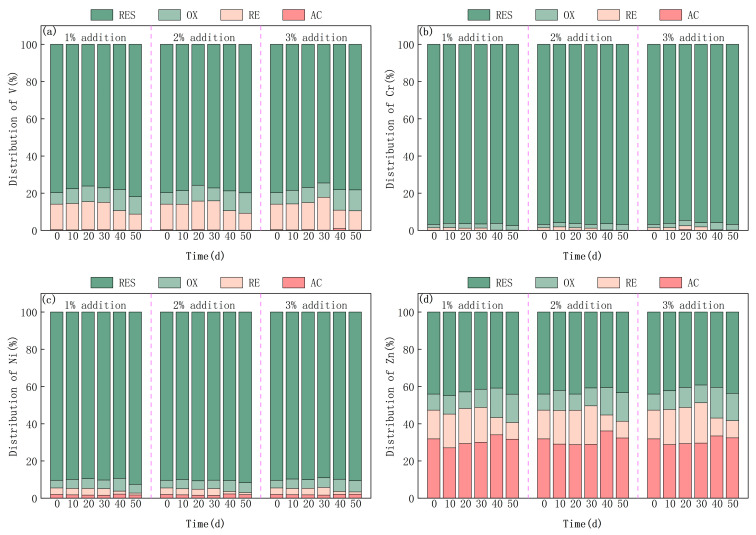
Changes in the chemical fractions of V (**a**), Cr (**b**), Ni (**c**), and Zn (**d**) over time after UiO-66-NH_2_ amendment. The day-0 values represent the initial untreated baseline before amendment addition.

In the untreated soil, V was mainly present in the residual fraction (79.67%), with weakly acid-extractable (0.35%) and reducible (13.77%) fractions contributing to potential migration risks (Figure 4a). After the addition of UiO-66-NH_2_, the speciation transformation significantly increased: at a 1% addition, the weakly acid-extractable and reducible fractions decreased by 42.77% and 38.03%, respectively, within 50 days, whereas the residual fraction only slightly increased by 2.77%, and the oxidizable fraction sharply increased by 51.24%. This result indicates that the active fractions were mainly transformed into oxidizable fractions rather than residual fractions. At 2% addition, the weakly acid-extractable and reducible fractions decreased by 43.35% and 34.41%, respectively, while the oxidizable and residual fractions increased by 76.51% and 0.17%, respectively, further confirming that the transformation is primarily blocked in the oxidizable state. At 3% addition, the weakly acid-extractable fraction decreased by 49.50%, reaching a peak, but the residual fraction unexpectedly decreased by 1.79% to 78.24%, while the oxidizable fraction increases to 80.35%. This may be due to the excessive passivation agent forming soluble complexes with V ions or the high concentration of the passivating agent increasing the solution viscosity, which hinders the diffusion of passivation molecules.

Regarding Cr (Figure 4b), its leaching concentration in the original soil was extremely low, with the residual fraction accounting for as high as 96.78%. This indicates that chromium primarily existed in the stable, low-bioavailability form of Cr (III). Following the addition of UiO-66-NH_2_, the leaching concentration of Cr decreased further. The highest inhibition rate of 85.71% was achieved after 50 days at a 3% application rate, demonstrating the material’s effectiveness in immobilizing potentially active chromium. At application rates of 1% and 2%, the weak acid-extractable fraction decreased by 6.67–11.48%, the reducible fraction was drastically reduced by over 95%, and the oxidizable fraction increased significantly by 37.77–64.88%. This transformation from active fractions to the oxidizable fraction suggests that UiO-66-NH_2_ likely functions through two synergistic mechanisms. First, its abundant surface amino groups can be protonated to form –NH_3_^+^ in the soil environment, enabling electrostatic adsorption of anionic Cr (VI). Second, the adsorbed Cr (VI) may be reduced to Cr (III) by the material itself or coexisting organic matter, subsequently complexing with functional groups such as carboxyl groups on the material surface, thereby transferring into oxidizable or residual fractions. However, at the high application rate of 3%, an anomalous increase of 2.05% was observed in the weak acid-extractable fraction. This may be attributed to localized acidification of the microenvironment caused by the excessive material, which could slightly mobilize a portion of acid-soluble Cr (III).

The transformation behavior of Ni and Zn was mainly governed by their cationic characteristics (Figure 4c,d). The original residual state of Ni accounts for 90.36%. At 1–2% additions, the weakly acid-extractable and reducible fractions decreased, and the residual state slightly increased; however, at 3% addition, the weakly acid-extractable fraction increased, and the residual fraction only slightly increased. This change may be related to the electrostatic repulsion between protonated –NH_2_ and Ni^2+^ and the competition for adsorption sites with Zn^2+^. For Zn, the active fraction accounts for 47.33%. At 1% addition, the reducible fraction sharply decreases by 41.97%, and the oxidizable fraction increased by 76.19%, whereas the residual fraction only slightly increased by 0.17%. At 2–3% additions, the weakly acid-extractable fraction unexpectedly increased, and the residual fraction decrease.

In conclusion, UiO-66-NH_2_ has a significant dose threshold effect on the stabilization of multimetal-polluted soils. At the optimal addition (1%), the overall inhibition rate of the active states (weak acid-extractable and reducible) of V, Cr, Ni, and Zn can exceed 38%, with V decreasing to 42.77% and Zn decreasing to 41.97%. However, excessive addition of a passivation agent increases the risk of reverse activation. For example, the residual state of V decreases by 1.79% at 3% addition, the weakly acid-extractable state of Cr increases by 2.05%, and the weakly acid-extractable fraction of Zn continuously increases at 2–3% addition. Based on these results, after the UiO-66-NH_2_ adsorbent was used, the order of ease of transformation from highly toxic, mobile states to more stable forms during soil adsorption was V > Ni > Cr > Zn. Overall, no single UiO-66-NH_2_ dosage was optimal for all four metals. The 1% treatment provided a practical performance-to-dosage compromise under laboratory incubation conditions because it effectively reduced the leaching of V, Ni, and Zn, whereas Cr required a higher dosage to achieve its maximum reduction efficiency. Therefore, the 1% treatment should not be interpreted as demonstrating field-scale cost-effectiveness. For the remediation of multi-metal-contaminated soils, it is crucial to strictly control the amount of passivating agent added to avoid weakening the stabilization effect due to metal interactions.

A preliminary material-cost comparison was added to evaluate the potential field applicability of UiO-66-NH_2_. Assuming the treatment of 1 t of dry soil, a 1% UiO-66-NH_2_ dosage requires approximately 10 kg of material. Based on reported techno-economic estimates for UiO-66-NH_2_ production, the material cost would be approximately 158–980 USD/t soil when aqueous and solvothermal synthesis costs are considered. By comparison, sepiolite, a conventional mineral stabilizer widely used for metal-contaminated soils, has a reported cost range of approximately 32–1000 USD/t depending on its application and processing level; at the same 1% dosage, this corresponds to approximately 0.32–10 USD/t soil. This comparison indicates that UiO-66-NH_2_ remains substantially more expensive than conventional mineral stabilizers at the current stage [32,33]. Thus, UiO-66-NH_2_ is more suitable as a high-performance functional amendment for targeted stabilization or mechanistic exploration, whereas large-scale in situ application would require further cost reduction through scalable synthesis, regeneration/reuse, or hybrid application with low-cost stabilizers.

### 3.3. Impact on Physicochemical Properties

As shown in Figure S1a, the soil pH generally decreased with increasing stabilization time under different UiO-66-NH_2_ dosages. At the 1% UiO-66-NH_2_ dosage, soil pH continuously decreased during the 50-day incubation period, with a final reduction of 0.67, corresponding to a 7.8% decrease. For the 2% and 3% treatments, soil pH also decreased over time, with final reductions of 0.60 and 0.62, respectively. These results indicate that UiO-66-NH_2_ addition altered the soil acid–base environment during stabilization. The pH variation provides useful mechanistic insight into metal speciation transformation. Because the final soil pH remained in the alkaline range, approximately 7.6, protonation of amino groups on UiO-66-NH_2_ should be regarded as one contributing mechanism rather than the only explanation. A slight decrease in soil pH may partly promote the formation of protonated –NH_3_^+^ sites and enhance electrostatic interactions with oxyanionic metal species such as vanadate and chromate. In addition, complementary mechanisms, including surface complexation with Zr–O/Zr–OH sites, coordination or ligand exchange at defect Zr sites, complexation between metal cations and N/O-containing functional groups, ion exchange associated with SOM and CEC changes, and physical adsorption within the porous UiO-66-NH_2_ structure, may jointly contribute to the immobilization of V, Cr, Ni, and Zn.

As shown in Figure S1b, the soil organic matter (SOM) slightly increased with increasing amounts of UiO-66-NH_2_ addition and longer treatment times. Compared with the initial value of 39.975 g/kg, the SOM content at 50 days increased by 0.024%, 0.095%, and 0.193% for the 1%, 2%, and 3% additions of UiO-66-NH_2_, respectively. With increasing addition amounts, the organic matter content in the soil increased, with the greatest increase occurring at the 3% addition, reaching 40.052 g/kg after 50 days. An increase in soil SOM improves soil fertility and the cation exchange capacity (CEC), buffers the soil, promotes plant growth, and supports microbial growth and metabolism. Moreover, humic acids in the soil, which are hydrophilic colloids, can absorb cations while forming a buffering system with salts, further increasing soil buffering capacity. The slight overall increase in SOM could be due to the passivation of various heavy metals in the soil by UiO-66-NH_2_, reducing heavy metal contamination and thus increasing microbial activity, which promotes the decomposition of organic carbon and nitrogen, leading to a slight increase in soil organic matter. In summary, the addition of UiO-66-NH_2_ increases the soil organic matter content, improves soil fertility, reduces the use of chemical fertilizers, and provides abundant functional groups (such as carboxyl and hydroxyl groups) in the organic matter, which act as adsorption sites. The increase in organic matter enhanced adsorption capacity and contributed to the stabilization performance of UiO-66-NH_2_.

Soil CEC plays an important role in the transformation of substances and solute transport in the soil environment, making it one of the key indicators for evaluating soil fertility and physicochemical properties [33]. Figure S1c shows that the soil CEC generally increases with increasing UiO-66-NH_2_ addition. However, with changes in treatment time, the soil CEC initially increased, then decreased, and finally increased again. During the first 20 days, the CEC increased continuously after 1%, 2%, and 3% UiO-66-NH_2_ was added. This may be due to the large surface area and abundant adsorption sites of the modified material, as well as the amino and hydroxyl groups on the surface, which promote the adsorption of heavy metal ions in the soil, increasing the CEC. Between 30 and 40 days, the cation content in the soil decreased below the initial value. This may be because UiO-66-NH_2_ adsorbs heavy metals to form stable compounds, approaching adsorption saturation, leading to a reduction in the free heavy metal ions in the environment, which results in a temporary decrease in the CEC. Between 40 and 50 days, the CEC increased slightly, likely because UiO-66-NH_2_ releases a small amount of adsorbed sites, enabling ion exchange, which enhances the CEC. It is also possible that the material reacts with certain organic substances or minerals in the soil, forming new adsorption sites, which increase the cation exchange capacity [34]. The results indicate that adding 2% and 3% UiO-66-NH_2_ increases soil CEC, enhancing the ability of the soil to adsorb cations and thereby improving the stabilization performance of the amendment toward heavy metals in soil.

### 3.4. Responses of Soil Microbial Diversity and Community Structure to UiO-66-NH_2_

Soil microorganisms play crucial roles in ecological processes and contribute to the maintenance of soil ecosystem functions [35]. The addition of stabilizers can alter the environmental properties of the soil to some extent, and microorganisms are often sensitive to these changes (Figure S2). Therefore, the distribution, structure, and diversity of soil microorganisms are important factors for evaluating soil ecosystems and serve as key indicators for assessing the effectiveness of stabilization remediation technologies [36]. Changes in these parameters can also indirectly reflect the degree of disturbance to the soil ecological environment caused by the remediation material.

As detailed in Table S2, soil amendment with UiO-66-NH_2_ altered the microbial community structure, characterized by a dose-responsive attenuation of alpha diversity indices. Compared to the control (CK), treatments T1–T3 showed a progressive decline in Richness, Shannon, and Chao1 values, with the Shannon index decreasing from 6.4289 to 4.2159 in T3. These observed trends suggest that the selective pressure from functionalized MOFs may have filtered out sensitive indigenous taxa while favoring resilient, metabolically versatile lineages. With sample coverage exceeding 0.99, the sequencing depth captured most of the soil phylotypes, confirming that this structural simplification is a robust response to MOF-induced environmental shifts.

**Figure 5:**
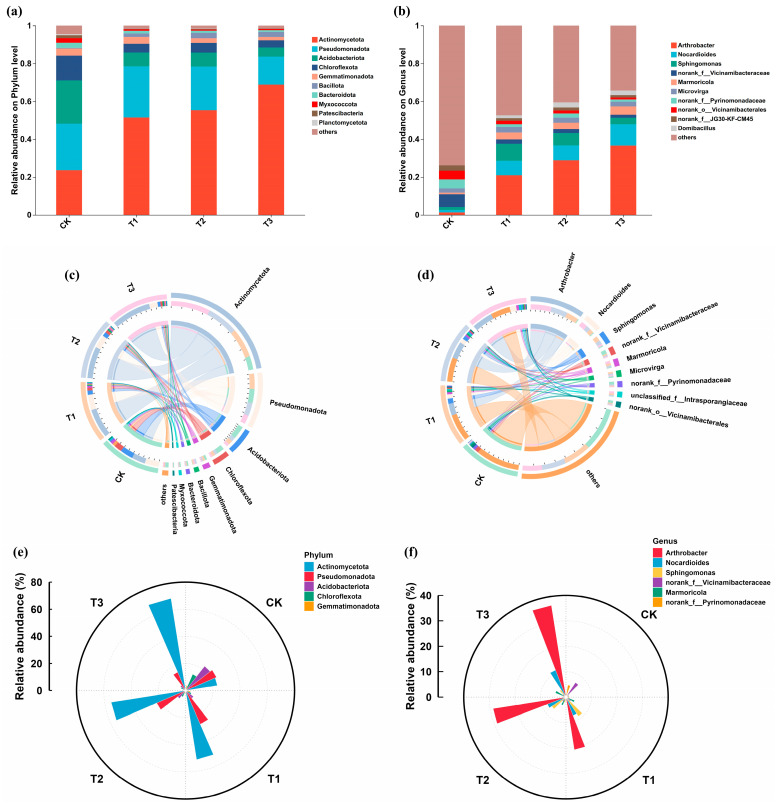
Changes in microbial community structure in contaminated soil under different UiO-66-NH_2_ amendment dosages. (**a**,**b**) Phylum and genus-level community structures before and after the experiment. (**c**,**d**) Phylum- and genus-level community structure circos diagram. (**e**,**f**) Relative abundance of key (UiO-66-NH_2_)-degrading genera.

The remediation process triggered a distinct successional shift (Figure 5). At the phylum level, The relative abundance of Actinomycetota increased and became dominant in T3, whereas Acidobacteriota and Chloroflexota—taxa typically thriving in stable, low-disturbance environments—were substantially decreased (Figure 5a,b). This polarization was even more pronounced at the genus level, where *Arthrobacter* increased from a low relative abundance in CK to the dominant genus in T3. Given the >85% immobilization of V, Cr, Ni, and Zn achieved in this study, the *Arthrobacter* bloom suggests that UiO-66-NH_2_ created a specific niche for taxa possessing high metal tolerance and metabolic plasticity.

The distribution of these taxa across treatments was highly asymmetric (Figure 5c,d). Sequence flow for Actinomycetota [24] was channeled almost exclusively toward the T1–T3 groups, and the extensive network connectivity between *Arthrobacter* and treated samples identifies it as a potential indicator for UiO-66-NH_2_ amendment. These patterns were characterized by the radial dominance of these groups peaking sharply in T3. Such targeted enrichment likely stems from the increased availability of surface amino (−NH_2_) groups and improved soil physicochemical properties—specifically SOM and CEC—which collectively provided optimized colonization sites for these specific functional groups [37].

All treatment groups branched independently from the control, identifying the MOF amendment as the primary driver of community drift. Functional genera such as *Arthrobacter*, *Domibacillus*, and *Ramlibacter* were enriched in the amended soils, contrasting with the conspicuous depletion of Acidobacteriota sub-groups (Figure 5e,f). The synchronous enrichment of diverse Actinomycetota genera suggests a synergistic niche expansion rather than a random shift. This directional restructuring of the microbial landscape is highly congruent with the observed reductions in metal bioavailability and the localized shifts in soil pH and organic matter content following remediation.

## 4. Conclusions

This study evaluated the stabilization performance of the amino-functionalized metal–organic framework UiO-66-NH_2_ for V–Cr–Ni–Zn multi-metal-contaminated soils in the Panzhihua mining area. Under the 1% UiO-66-NH_2_ treatment at 50 days, the leaching concentrations of V, Cr, Ni, and Zn decreased by 90.42%, 59.72%, 90.12%, and 90.71%, respectively. Although the 3% treatment showed the highest Cr reduction efficiency of 85.71%, no single dosage was optimal for all four metals. Therefore, the 1% treatment should be regarded as a practical compromise rather than a universal optimum, because it achieved strong immobilization of V, Ni, and Zn while maintaining a relatively low amendment dosage. The overall inhibition rate of the active states (weak acid-extractable and reducible fractions) exceeded 38%, with the active fraction of V decreasing by 42.77% and the reducible fraction of Zn decreasing by 41.97%. The core mechanism involved driving the transformation of heavy metals from active states to oxidizable states, with the proportion of oxidizable V, Cr, and Ni increasing by 37.8–80.4%, effectively reducing their bioavailability and converting short-term environmental risks into long-term slow-release forms.

In addition to metal immobilization, the amendment improved soil ecological functions by enhancing physicochemical properties and shaping the microbial community. The addition of UiO-66-NH_2_ increased SOM and CEC, thereby creating more favorable conditions for microbial activity. Together with the observed pH changes, these physicochemical variations helped explain the different stabilization responses of V, Cr, Ni, and Zn. Microbial profiling showed that the material exerted a selective pressure on the community, leading to a dose-dependent decrease in alpha diversity. The community structure shifted toward more tolerant taxa, with Actinomycetota at the phylum level and *Arthrobacter* at the genus level becoming dominant. These microbial results provide preliminary evidence that UiO-66-NH_2_ amendment may influence soil ecological responses during stabilization. However, as the microbial community patterns were mainly interpreted based on descriptive trends, future studies should incorporate more comprehensive statistical analyses, including ANOVA or Kruskal–Wallis tests for α-diversity indices and PERMANOVA for β-diversity, to further verify the significance of microbial community differences among treatments. The novelty of this study lies in the integrated evaluation of UiO-66-NH_2_-mediated metal immobilization, chemical fraction transformation, soil physicochemical changes, and microbial community responses in a complex multi-metal-contaminated soil matrix. These findings suggest that UiO-66-NH_2_ has potential as a functional amendment for the stabilization of multi-metal-contaminated mining soils. However, although UiO-66-NH_2_ showed promising stabilization performance in this laboratory study, its current material cost remains a major limitation for direct large-scale in situ application. Future work should focus on cost reduction, scalable synthesis, and combined application with low-cost conventional stabilizers.

## Data Availability

The original contributions presented in this study are included in the article/Supplementary Materials. Further inquiries can be directed to the corresponding author.

## Supplementary Materials

The following supporting information can be downloaded at: https://www.mdpi.com/article/10.3390/toxics14060462/s1, Table S1: Analysis of statistical characteristics of soil heavy metal content in the study area; Table S2: Microbial community diversity index; Figure S1: (a) Effects on pH of contaminated soil; (b) Effects on SOM; (c) Effects on CEC; Figure S2: Microbial community structure heatmap; Table S3: The change in V leaching concentration in soil under different passivator dosage; Table S4: The change in Cr leaching concentration in soil under different passivator dosage; Table S5: The change in Ni leaching concentration in soil under different passivator dosage; Table S6: The change in Zn leaching concentration in soil under different passivator dosage; Table S7: The change in chemical form ratio of V in soil under different passivator dosage; Table S8: The change in chemical form ratio of Cr in soil under different passivator dosage; Table S9: The change in chemical form proportion of Ni in soil; Table S10: The change in chemical form proportion of Zn in soil. Additional information has been provided in the supplementary documents.

## Abbreviations

The following abbreviations are used in this manuscript:

SOM	Soil organic matter
CEC	Cation exchange capacity
