# Supplementary material for: Application of Amino-Functionalized Metal–Organic Framework UiO-66-NH2 in the Remediation of Multi-Metal-Contaminated Soil in Mining Areas
Source: Toxics. 2026 May 25;14(6):462. doi: 10.3390/toxics14060462 (PMC13306549; doi:10.3390/toxics14060462)
Supplement: Supplementary file 1 [file toxics-14-00462-s001.zip › toxics-4276410-supplementary.pdf]

Table S1. Analysis of statistical characteristics of soil heavy metal content in the study area.

Element	V	Cr	Ni	Zn
Maximum (mg/kg)	1304.44	814.44	122.22	546.67
Minimum (mg/kg)	88.45	44.42	26.89	48.06
Average (mg/kg)	382.42	210.56	62.86	235.46
Standard deviation (mg/kg)	280.40	166.49	22.72	149.02
Coefficient of variation (%)	73.32	79.07	36.14	63.29
Background value of soil heavy metals(SiChuan) (mg/kg)	92.60	79.00	32.60	86.50

Table S2. Microbial community diversity index.

SampleID	Richness	Shannon	Simpson	Chao1	Coverage
CK	2165	6.4289	0.9959	2210.6422	0.9951
T1	1724	5.0385	0.9529	1800.446	0.9937
T2	1634	4.6521	0.9241	1737.6885	0.9932
T3	1645	4.2159	0.8838	1817.1521	0.991

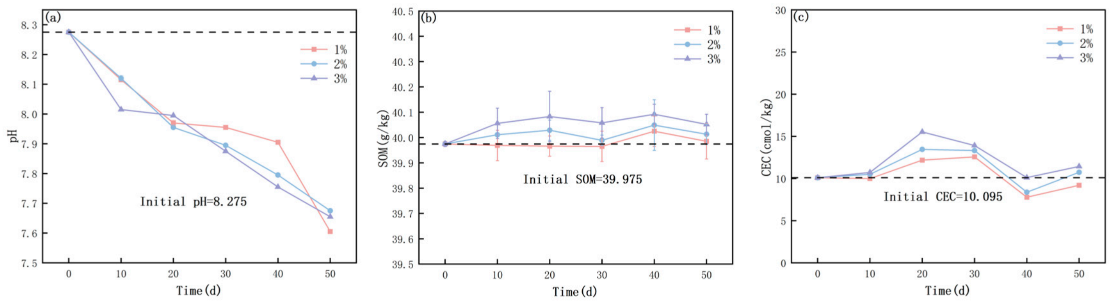

Figure S1. (a) Effects on pH of contaminated soil; (b) Effects on SOM; (c) Effects on CEC.

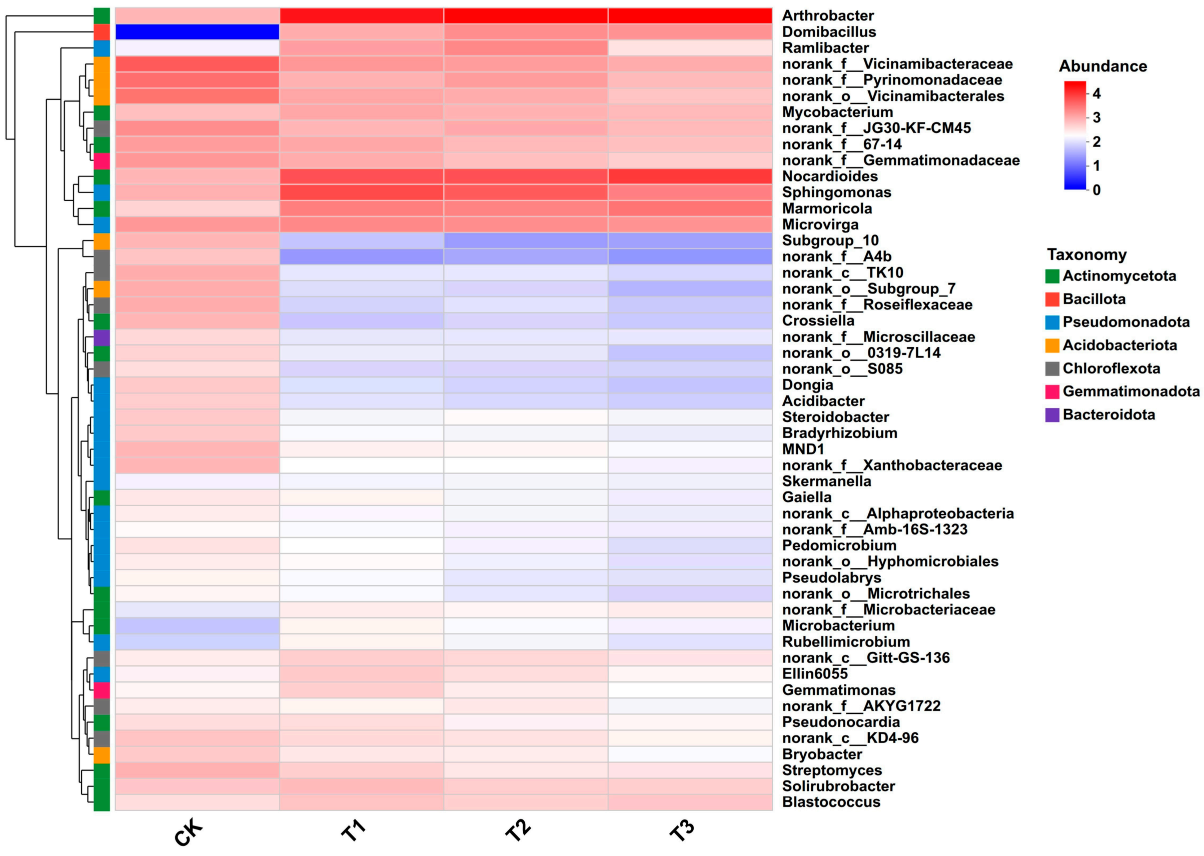

Figure S2. Microbial community structure heatmap.

Table S3. The change of V leaching concentration in soil under different passivator dosage.

initial concentration(mg/kg)	days	1% passivator leaching concentration (mg/kg)	2% passivator leaching concentration (mg/kg)	3% passivator leaching concentration (mg/kg)
	0	4.645	4.645	4.645
4.645	10	0.32757	0.27981	0.345375
4.645	20	0.39678	0.348175	0.43835
4.645	30	0.499985	0.55147	0.685885
4.645	40	0.636525	0.40417	0.499455
4.645	50	0.445	0.55403	0.476025

Table S4. The change of Cr leaching concentration in soil under different passivator dosage.

initial concentration(mg/kg)	days	1% passivator leaching concentration (mg/kg)	2% passivator leaching concentration (mg/kg)	3% passivator leaching concentration (mg/kg)
	0	0.043445	0.043445	0.043445
0.043445	10	0.0249	0.018315	0.015465
0.043445	20	0.01478	0.010265	0.01864
0.043445	30	0.01776	0.01235	0.010725
0.043445	40	0.016065	0.008285	0.007615
0.043445	50	0.0175	0.01056	0.00621

Table S5. The change of Ni leaching concentration in soil under different passivator dosage.

initial concentration(mg/kg)	days	1% passivator leaching concentration (mg/kg)	2% passivator leaching concentration (mg/kg)	3% passivator leaching concentration (mg/kg)
	0	0.17711	0.17711	0.17711
0.17711	10	0.14172	0.097635	0.12014
0.17711	20	0.07318	0.06806	0.097175
0.17711	30	0.06788	0.050495	0.08403
0.17711	40	0.03052	0.04047	0.058815
0.17711	50	0.01738	0.02018	0.01791

Table S6. The change of Zn leaching concentration in soil under different passivator dosage.

initial concentration(mg/kg)	days	1% passivator leaching concentration (mg/kg)	2% passivator leaching concentration (mg/kg)	3% passivator leaching concentration (mg/kg)
	0	1.62382	1.62382	1.62382
1.62382	10	1.20119	0.78161	1.3259
1.62382	20	0.43334	0.37408	1.05912
1.62382	30	0.246925	0.346705	1.148865
1.62382	40	0.175915	0.43835	0.9654
1.62382	50	0.15097	0.31571	0.20059

Table S7. The change of chemical form ratio of V in soil under different passivator dosage.

passivator dosage (%)	days	AC(%)	RE(%)	OX(%)	RES(%)
1	0	0.35343	13.76943	6.21517	79.66197
1	10	0.26808	14.19373	7.92747	77.61072
1	20	0.42529	15.05234	8.36195	76.16042
1	30	0.34066	14.72467	7.83005	77.10462
1	40	0.25357	10.33454	11.37583	78.03606
1	50	0.20225	8.53233	9.40003	81.86539
2	0	0.35343	13.76943	6.21517	79.66197
2	10	0.21098	13.84202	7.32275	78.62425
2	20	0.43252	15.30848	8.50425	75.75475
2	30	0.26029	15.61252	6.97108	77.15611
2	40	0.14787	10.45593	10.61548	78.78072
2	50	0.20022	9.03077	10.97068	79.79833
3	0	0.35343	13.76943	6.21517	79.66197
3	10	0.21361	14.02266	7.30167	78.46207
3	20	0.4109	14.54303	8.07902	76.96706
3	30	0.20705	17.61136	7.65949	74.5221
3	40	0.94387	9.9597	10.91699	78.17944
3	50	0.17847	10.3763	11.20925	78.23597

Table S8. The change of chemical form ratio of Cr in soil under different passivator dosage.

passivator dosage (%)	days	AC(%)	RE(%)	OX(%)	RES(%)
1	0	0.09986	1.30918	1.80784	96.78311
1	10	0.07684	1.36367	2.41449	96.14501
1	20	0.054	1.18747	2.34343	96.41511
1	30	0.07019	1.19097	2.22407	96.51477
1	40	0.11473	0.07326	3.57063	96.24138
1	50	0.0932	0.06078	2.4907	97.35532
2	0	0.09986	1.30918	1.80784	96.78311
2	10	0.07038	1.80596	2.31	95.81366
2	20	0.19038	1.21454	2.30479	96.29029
2	30	0.05456	1.17863	2.01913	96.74768
2	40	0.17301	0.06375	3.3312	96.43205
2	50	0.0884	0.06352	2.98069	96.86739
3	0	0.09986	1.30918	1.80784	96.78311
3	10	0.06554	1.38682	2.29909	96.24856
3	20	0.42908	2.06623	2.90755	94.59715
3	30	0.06473	1.82206	2.27631	95.8369
3	40	0.22955	0.07709	3.91171	95.78165
3	50	0.10191	0.06609	3.0407	96.7913

Table S9. The change of chemical form proportion of Ni in soil.

passivator dosage (%)	days	AC(%)	RE(%)	OX(%)	RES(%)
1	0	1.96717	3.53476	4.13668	90.36139
1	10	1.79504	3.46012	4.87351	89.87133
1	20	1.63657	3.5775	5.26955	89.51638
1	30	1.59497	3.5395	4.61533	90.2502
1	40	2.17851	1.59416	6.89494	89.33239
1	50	1.69388	1.02956	4.70721	92.56935
2	0	1.96717	3.53476	4.13668	90.36139
2	10	1.77301	3.30855	4.98764	89.9308
2	20	1.5927	3.20225	4.51752	90.68752
2	30	1.47868	3.6872	4.51471	90.31941
2	40	2.25524	1.34133	5.90303	90.5004
2	50	1.91087	1.07683	5.35126	91.66104
3	0	1.96717	3.53476	4.13668	90.36139
3	10	1.81578	3.38445	5.05882	89.74095
3	20	1.75591	3.5699	4.58236	90.09183
3	30	1.64521	4.12137	5.21099	89.02243
3	40	2.06503	1.50889	6.57993	89.84616

Table S10. The change of chemical form proportion of Zn in soil.

passivator dosage (%)	days	AC(%)	RE(%)	OX(%)	RES(%)
1	0	31.90212	15.43357	8.69169	43.97262
1	10	27.08061	18.16475	9.95749	44.79715
1	20	29.40465	18.86687	8.8884	42.84008
1	30	30.07649	18.76882	9.7469	41.40779
1	40	34.10824	9.21784	15.87686	40.79706
1	50	31.68519	8.95545	15.31393	44.04543
2	0	31.90212	15.43357	8.69169	43.97262
2	10	29.07332	18.17506	10.73521	42.01641
2	20	28.80897	18.48467	8.70834	43.99802
2	30	29.00034	20.69141	9.58712	40.72114
2	40	36.18537	8.55555	14.72639	40.53269
2	50	32.38705	9.02379	15.33193	43.25722
3	0	31.90212	15.43357	8.69169	43.97262
3	10	28.85765	18.86497	10.02082	42.25656
3	20	29.37023	19.44653	10.80099	40.38225
3	30	29.62635	21.68272	9.57312	39.11782
3	40	33.48348	9.58999	16.59608	40.33045
3	50	32.45553	9.21912	14.77793	43.54742
